# Resistin, Visfatin, Adiponectin, and Leptin: Risk of Breast Cancer in Pre- and Postmenopausal Saudi Females and Their Possible Diagnostic and Predictive Implications as Novel Biomarkers

**DOI:** 10.1155/2015/253519

**Published:** 2015-03-08

**Authors:** Adel M. A. Assiri, Hala F. M. Kamel, Mohamed F. R. Hassanien

**Affiliations:** ^1^Biochemistry Department, Faculty of Medicine, Umm Al-Qura University, Makkah, Saudi Arabia; ^2^Medical Biochemistry Department, Faculty of Medicine, Ain Shams University, Cairo, Egypt; ^3^Institute of Scientific Research and Revival of Islamic Heritage, Umm Al-Qura University, Makkah, Saudi Arabia

## Abstract

The mechanisms of obesity-induced breast carcinogenesis are not clear. One hypothesis is that high levels of adipokines could promote breast cancer (BC) development. The aim of this study was to investigate the correlation of resistin, visfatin, adiponectin, and leptin with BC risk in pre- and postmenopausal females. A total of 82 BC newly diagnosed and histologically confirmed patients and 68 age and BMI matched healthy controls were enrolled. Both groups were subdivided into post- and premenopausal subgroups. Resistin, visfatin, adiponectin, and leptin were measured by ELISA. There were significantly higher levels of leptin, resistin, and visfatin in postmenopausal BC patients than their respective controls. Only in postmenopausal subgroups, leptin, resistin, and visfatin levels were positively correlated with TNM staging, tumor size, lymph node (LN) metastasis, and histological grading. In postmenopausal females, multivariate logistic regression analysis revealed that adiponectin, leptin, visfatin, and resistin were risk factors for BC. Our results suggested that serum resistin, leptin, adiponectin, and visfatin levels as risk factors for postmenopausal BC may provide a potential link with clinicopathological features and are promising to be novel biomarkers for postmenopausal BC.

## 1. Introduction 

Studies indicated that obesity, as reflected by increased body mass index (BMI), is associated with increased risk of more aggressive BC [1, 2]. Obese women are likely to have metastatic BC when they are first diagnosed and to have a poor prognosis regardless of their menopausal status [3, 4]. Accumulating evidence suggests that adipose tissue, as an endocrine organ producing and secreting a large range of factors, may interfere with cancer development. These factors, called adipokines, involved in the mediation of inflammatory diseases and obesity [5, 6]. Adipokines, as leptin, adiponectin, visfatin, and resistin, are produced by different fat depots, including subcutaneous, visceral, and mammary adipose tissues. It is worth noting that adipokines may act on breast tissue in an endocrine manner, in a paracrine pathway, and in an autocrine action [7, 8]. The structure of the mammary gland may be in favor of close interaction between mammary adipose tissue and breast tissue, which suggests that adipokines produced by mammary adipose tissue and the tumor cell microenvironment may be the major link between obesity and BC progression and metastasis [9, 10]. 

Obesity has effects on a number of hormones and growth factors potentially linked to BC. A major cluster of obesity-related metabolic consequences include altered concentrations of circulating adipocytokines and development of insulin resistance including hyperinsulinaemia and impaired glucose metabolism [11]. The most prominent adipokine, leptin, was first described as a neurohormone whose primary function is to regulate energy balance and food intake in the hypothalamus. Subsequent studies found that leptin can modulate several processes in the peripheral organs, such as immune response, fertility, and hematopoiesis. On a cellular level, leptin has been found to act as a mitogen, metabolic regulator, motogenic, and proangiogenic factor [12]. New evidence suggests that leptin could be involved in tumorigenesis, especially in the development of breast, colorectal, and prostate cancers [13]. In several BC cell models, leptin has been shown to induce proliferation, survival, and anchorage-independent growth. These leptin activities are mediated through the long/signaling form of the leptin receptor (ObRL) that, upon leptin binding, can stimulate the Jak/STAT3, ERK1/2, and phosphoinositide 3-kinase pathways as well as inducing cyclin D1 expression and retinoblastoma protein hyperphosphorylation [14, 15]. Adiponectin is another adipocyte-derived peptide hormone that is inversely associated with adiposity [16]. Adiponectin is a strong indicator of insulin sensitivity wherein its decline precedes the onset of obesity and insulin resistance [17] and may be one mechanism through which obesity alters BC risk. Mantzoros et al. [18] found an inverse association between serum adiponectin levels and BC risk among postmenopausal women.

Resistin, named for resistance to insulin, is a unique signaling molecule secreted from adipocytes. Resistin may serve as a hormone that potentially links obesity to insulin resistance [19]. High level of resistin was reported to be associated with the risk of BC, wherein this relationship was independent of age, BMI, status of menopause, serum glucose, and adiponectin [20]. Visfatin, also known as pre-B cell colony-enhancing factor, is a novel adipokine, found in the visceral fat. Visfatin plays an important role in a variety of metabolic and stress responses as well as in the cellular energy metabolism as Nampt (nicotinamide phosphoribosyltransferase) [21]. Interestingly, it was shown that serum visfatin is significantly elevated in patients with gastric carcinoma and BC and may be a promising biomarker for colorectal adenocarcinoma [22]. High expression of visfatin in BC tissues was reported to be associated with more malignant cancer behavior as well as adverse prognosis [23]. Epidemiologic studies have demonstrated that age, hormone-associated reproductive factors such as earlier age at menarche, later age at menopause, anthropometric characteristics such as BMI, and waist circumference (WC) are well-established risk factors for BC [24, 25].

Recent studies have shown that the constellation of obesity, insulin resistance, and serum adipocytokine levels is associated with the risk and prognosis of BC, especially postmenopausal breast cancer (PBC) [26, 27]. However, the exact mechanisms responsible for the complexity of the effect of obesity and various adipokines on BC risk in pre- and postmenopausal women need to be elicited. We aimed in our study to investigate the correlation of resistin, visfatin, adiponectin, and leptin with BC risk in pre- and postmenopausal females and to evaluate their relation to other metabolic, anthropometric measures with different clinicopathological features of BC.

## 2. Materials and Methods 

### 2.1. Study Populations

Population enrolled in this study was 150 Saudi females. BC patients (*n* = 82) were newly diagnosed and histologically confirmed BC with no prior surgical, chemotherapy, or radiotherapy BC treatment, who attended King Abdulla Medical City or El-Noor Hospital (Makkah, KSA) from March 2011 till December 2013. Healthy control subjects (*n* = 68) were age and BMI matched with BC group. Both groups were subdivided into two subgroups according to menopausal status. Women (*n* = 41, healthy control and 38 cases) were considered postmenopausal at the time of blood collection (if they had no menstrual cycles in the last 12 months). Women (*n* = 44, cases and 27 controls) were defined as premenopausal at blood draw (if reported that their periods had not ceased and were 45 years or younger). Once patients and control subjects agreed to participate, written informed consent was obtained. For all subjects, comprehensive questionnaires were used to collect medical information. Complete history was obtained including menstrual and reproductive history, menopausal status, lifestyle behaviors, and medical history as well as family history of BC and other cancers. Because questionnaire data and specimens were obtained prior to cases' acceptance with surgery and radio- or chemotherapy, any influence of treatment was unlikely. All cases and controls who participated in our study were fully informed of the aim of the study and gave written consent for their participation. The study was approved by the Faculty of Medicine Ethics Review Board for Human Studies at Umm Al-Qura University (Makkah, KSA).

### 2.2. Anthropometric Measurements and Clinical Examination

For all participants physical, clinical examinations and anthropometric measurements were performed. Height (to the nearest 0.5 cm) and weight (to the nearest 0.1 kg) were measured. BMI was calculated as weight in kg divided by the square of height in meters. Waist circumference was measured using a standardized measuring tape in cm, in addition to systolic and diastolic blood pressure measurements. All control subjects were confirmed free from benign or malignant breast diseases by physical examination and mammography. Women with history or family history of any tumor or cancer were excluded. Diagnosis of BC was confirmed histologically in each case and estrogen receptor statues were determined. The staging of BC was determined according to the TNM system.

### 2.3. Laboratory Measurements

Blood samples were collected from study subjects after overnight fasting for 12 h using BD vacutainer serum tubes. Samples were transported in portable insulated bags containing ice packs (0–4°C) and processed by centrifugation at 1467 g for 10 min at room temperature within 6 h of collection. Plasma, buffy coat, and red blood cells were separated and stored at −70°C until subsequent analysis. The concentration of leptin (ng/mL) was determined using ELISA kits (Millipore, Missouri, USA). The concentration of total adiponectin (ng/mL) was determined using sandwich ELISAs with kits (ALPCO Diagnostics, North Carolina, USA). The total adiponectin values in this study represented measurements of trimer, hexamer, and high molecular weight (HMW) forms of adiponectin in blood. Serum visfatin levels (ng/mL) were measured using a commercial enzyme immunoassay kit (Phoenix Pharmaceuticals, Belmont, CA, USA). Resistin (*μ*g/L) was determined by immunosorbent assays (ELISA, R&D systems Inc., USA). All samples were assayed in duplicate and completely blinded to the clinical information. All procedures in the manufacturer's instructions were followed, and quality control measurements were within the ranges recommended by the manufacturer. The minimum detectable concentration for the leptin kit was 0.25 ng/mL, and its intra-assay and interassay coefficients of variation (CVs) ranged from 3.0% to 6.2%. The minimum detectable concentration for the adiponectin kit was 0.15 ng/mL, wherein its intra-assay and interassay coefficient of variation (CVs) ranged from 2.9% to 6.6%. The minimum detectable concentration for visfatin assay was 0.1 ng/mL, with an intra-assay coefficient of variation 5.2% and an interassay coefficient of variation 5.8%. The intra- and interassay coefficients of variation were 5.2% and 7.8% for resistin. Fasting blood glucose (mg/dL) was determined using a glucose oxidase assay and the serum concentrations of LDLc (mg/dL). Total cholesterol TC in mg/dL, HDLc (mg/dL), and triglycerides (mg/dL) were determined using colorimetric enzyme kits (Spinreact, Bas Gerona, Spain).

### 2.4. Statistical Analysis

Data were analyzed with the IBM Statistical Package for the Social Sciences SPSS version 20.0. Descriptive data were given as mean ± standard deviation (SD). Comparison between the two groups was performed with *t*-test and covariance analysis, whereas comparisons among subgroups were performed using ANOVA test. Post hoc pairwise comparisons were performed using the Bonferroni method. The Pearson correlation coefficients (*r*) were used as measurements of correlation for continuous normally or not normally distributed variables, respectively. The relationships clinic-pathological characters of BC cases (TNM, tumor size, LN, and histological grade) and variables were analyzed by linear correlation and stepwise regression. The logistic multivariant regression analysis was used to analyze the relationship between adipocytokines, metabolic factors, and risk of LN metastasis of BC. *P* value less than 0.05 was considered statistically significant.

## 3. Results 

The baseline characteristics of all subjects are summarized in Table 1. The mean age (±SD) of the cases and controls was 53.68 and 52.25 years, respectively, and there was no statistical difference between them (*P* = 0.567). BC patients showed higher WC than control subjects (*P* = 0.026). Meanwhile, systolic and diastolic blood pressure showed no significant difference between both groups (*P* = 0.096 and 0.147), respectively.There were higher levels of metabolic parameters including TG, TC, and LDLc among BC patients than controls (*P* = 0.000), while HDLc levels were significantly lower in controls than cancer patients (*P* = 0.000).

Serum levels of leptin, resistin, and visfatin were significantly higher in BC patients than controls (*P* = 0.000), while adiponectin levels were significantly low (*P* = 0.000) in BC patients. Interestingly, we found no significant difference between premenopausal BC patients and premenopausal controls for leptin, adiponectin, resistin, and visfatin (*P* = 0.228, 0.59, 0.52, and 0.85, resp.). Meanwhile, we found significantly higher levels of leptin, resistin, and visfatin (*P* = 0.000) in postmenopausal BC patients than postmenopausal normal controls. Serum adiponectin levels were significantly lower in postmenopausal BC patients than respective controls (*P* = 0.000). Anthropometric measurements showed that WC levels were significantly higher in postmenopausal BC patients than postmenopausal control (*P* = 0.00) and no significant difference was found between premenopausal BC and premenopausal control groups (*P* = 0.50) as shown in Tables 1 and 2.

Pearson's correlation analysis using anthropometric, metabolic, and adipokines levels among all groups (*n* = 150) and healthy control (*n* = 68) revealed very interesting finding. When applying the test among healthy control group, the studied adipokines levels showed non significant correlation with other metabolic or anthropometric measurements as shown in Table 3. Data reveled that among all groups serum adiponectin levels showed negative correlation with BMI, WC, and TG (*r* = −0.232, *P* = 0.004; *r* = −0.295, *P* = 0.000; and *r* = −0.337, *P* = 0.000, resp.) and positive correlation with HDLc (*r* = 0.216, *P* = 0.008). Serum leptin levels were positively correlated to BMI, WC, DBP, glucose, and TG (*r* = 0.313, *P* = 0.000; *r* = 0.276, *P* = 0.001; *r* = 0.187, *P* = 0.022; *r* = 0.242, *P* = 0.003; and *r* = 0.176, *P* = 0.031, resp.) but negatively with HDLc (*r* = −0.170, *P* = 0.38). Serum resistin concentrations were positively correlated to SBP, TG, and TC (*r* = 0.192, *P* = 0.019; *r* = 0.185, *P* = 0.023; and *r* = 0.316, *P* = 0.000, resp.) but negatively to HDLc (*r* = −0.281, *P* = 0.000). Serum visfatin levels showed positive significant correlations with BMI, WC, TG, and glucose (*r* = 0.288, *P* = 0.005; *r* = 0.259, *P* = 0.001; *r* = 0.283, *P* = 0.000; and *r* = 0.182, *P* = 0.026). All the studied adipokines showed no significant correlations with the previous metabolic and anthropometric measurements among healthy control group (*P* > 0.05).

Table 4 depicts the correlations of serum adiponectin, leptin, resistin, and visfatin levels with clinicopathological features of pre- and postmenopausal BC patients. Leptin levels were positively correlated with TNM, tumor size, LN metastasis, and histological grading (*r* = 0.338, *P* = 0.038; *r* = 0.241, *P* = 0.029; *r* = −0.820, *P* = 0.000; and *r* = −0.762, *P* = 0.000). Serum resistin levels also were positively correlated with TNM, tumor size, LN metastasis, and histological grading (*r* = 0.395, *P* = 0.014; *r* = 0.429, *P* = 0.007; *r* = 0.575, *P* = 0.000; and *r* = 0.509, *P* = 0.000). Serum visfatin showed positive correlations with TNM, tumor size, and LN metastasis (*r* = 0.406, *P* = 0.000; *r* = 0.348, *P* = 0.028; and *r* = 0.529, *P* = 0.005). On the other hand, serum adiponectin levels correlated negatively with all clinicopathological (*P* < 0.05) features and *r* and *P* values were shown in Table 4.

Among postmenopausal group multivariate logistic regression analysis, with the risk for BC as the dependent variable and adiponectin, leptin, resistin and visfatin, BMI, WC, TG, TC, HDLc, LDLc, and glucose as independent variables, showed that the variables including BMI, TG, adiponectin, leptin, visfatin, and resistin were accepted by the final model. The final model suggested that BMI, TG, adiponectin, leptin, visfatin, and resistin were risk factors for BC, and the OR for BMI, TG, adiponectin, leptin, visfatin, and resistin was 0.370 (95% CI: 1.004–4.278, *P* = 0.049), 6.011 (95% CI: 1.823–15.673, *P* = 0.004), 0.423 (95% CI: 0.132–0.692, *P* = 0.005), 0.244 (95% CI: 1.381–3.587, *P* = 0.001), 1.089 (95% CI, 1.062–1.116; *P* = 0.001), and 0.513 (95% CI: 3.051–22.790, *P* = 0.000), respectively (Table 5). In addition, the dependent variable was the risk for lymph node status (positive versus negative) and the same independent variables mentioned above were employed as independent variables. Resistin, leptin, and visfatin were accepted by the final model and the OR for them was 2.203 (95% CI: 4.091–20.791, *P* = 0.002), 0.742 (95% CI: 1.504–2.921, *P* = 0.003), and 1.080 (95% CI: 1.056–1.105; *P* = 0.003), respectively (Table 6 ).

## 4. Discussion 

BC is one of the most common cancers and a leading cause of cancer death in women worldwide. BC is a disease of aging and thus is more common in postmenopausal versus premenopausal women. There is substantial evidence that the risk of postmenopausal BC is increased for postmenopausal obese women [28]. Analyses of the recent data from a large number of cases revealed that higher body weight was linked with the occurrence of hormone receptor-positive tumors [29, 30]. Obesity is an established risk factor for most hormone dependent cancers, including BC. The pathology underlying this phenomenon may be related to the endocrine and metabolic profile of this state. Recently researchers try to understand the link and underlying connection between obesity and BC. Studies focusing on adipocytokines and BC etiopathogenesis have shown that alterations in adipocytokines affect cell proliferation, apoptosis, tumor invasion, and angiogenesis [16]. In this study, we determined serum levels of leptin, adiponectin, resistin, and visfatin and we investigated the relation of those adipokines with other metabolic and anthropometric measurements to probe their relationship with risk of BC and other clinicopathological features. We also investigated the predictive role of those adipokines as regards their potential correlation and with clinicopathological features and anthropometric and metabolic parameters. Our results identified that there were statically higher serum levels of leptin, visfatin, and resistin and lower serum level of adiponectin in breast cancer patients compared to healthy controls.

Besides we demonstrated significant increase of leptin, visfatin, and resistin but significant decrease in adiponectin in postmenopausal BC when compared with postmenopausal control. There was also slight increase in levels of leptin, visfatin, and resistin in premenopausal BC cases in comparison to premenopausal controls. Regarding adiponectin levels, adiponectin levels were significantly lower only in postmenopausal BC not in premenopausal cancer subgroup compared to respected controls. Those findings were in agreement with other reports [31, 52]. To date, the specific mechanism by which postmenopausal women, but not premenopausal women, have an obesity-related risk of BC has not been clarified. However, some studies mentioned the potential involvement of estrogens. It is well documented that the production of estrogen between premenopausal and postmenopausal women differs significantly. The main source of estrogen production in premenopausal women is the ovaries, whereas, for postmenopausal women, it is from peripheral adipose tissue [27, 33]. In postmenopausal women, fat cells aromatize ovarian and adrenal androgens into estrogen. It has been demonstrated that elevated circulating levels of free estrogen, as well as estrone and androgens, are associated with BC [34]. Our data showed significantly elevated mean levels of serum TG, TC, LDLc, and reduced HDLc in all BC cases compared to healthy controls. Glucose levels were higher but not to significant value. Previous studies reported that high fasting glucose levels were directly correlated with BC both in premenopausal and postmenopausal women [35, 36]. In addition, reduced HDLc and increased blood pressure contributed to increased risk for BC [37, 38]. Furthermore, it has been reported that low HDLc, hypertension, and hyperglycemia have all been associated with BC [35, 37, 39, 40].

We studied the association of different adipokines, metabolic as well as anthropometric parameters, and the risk of BC. Interestingly, only in postmenopausal subgroups we found significant associations between elevated levels of leptin, resistin, and visfatin, BMI, and TG of developing BC. Such association could not be found in premenopausal females. On the other hand, adiponectin had a protective effect with the risk of developing BC only in postmenopausal females. Previously, obesity was reported as a risk factor for postmenopausal BC [4, 22, 41–44], whereas, in premenopausal women there is an inverse relation between BMI and BC risk [45, 46]. Their results have indicated that this association may be partly explained by the high levels of circulating estrogen of their results for obese women. The adipose tissue of postmenopausal obese women secretes more biologically active estrogen which may stimulate mammary epithelial cell mitosis and promote the development of tumor [47]. Adipose tissue including intratumoral sites in breast tissue expresses aromatase enzyme that may play an important role in postmenopausal BC progression in relation to obesity status. It has been reported that hyperleptinemia enhanced aromatase activity resulting in increased synthesis of estrogens [48, 49]. Leptin also has been reported to upregulate the expression of estrogen receptor-*α* (ER*α*) [50]. In contrast to our finding in premenopausal subgroups, a significant positive association between plasma leptin levels and premenopausal BC risk was reported [51]. Conversely, other retrospective studies have reported nonsignificant positive associations between leptin levels and premenopausal BC risk [32, 52, 53]. However, another study reported no association with premenopausal BC [54]. These conflicting results may partly be explained by the measurement of leptin after diagnosis, variation in sample sizes, varying level of control for confounders, differences in sample collection and measurement techniques, and heterogeneity of associations with pre- and postmenopausal BC.

We demonstrated that leptin, resistin, and visfatin might increase the risk of onset and lymph node metastasis of postmenopausal BC cases only and not in premenopausal BC group. In agreement with our results, Hou et al. [52] indicated that hyperleptinemia may be associated with BC especially with the onset and metastasis of BC after menopause, suggesting that hyperleptinemia may mediate the relationship between obesity and BC. Leptin has been reported to enhance aromatase activity in MCF-7 cell lines [55] which may enhance estrogen production and induce tumor cell growth [56]. Similarly, leptin receptors are expressed in T47-D BC cell lines and leptin induces proliferation of T47-D cells [57, 58]. However, leptin may have the potential to reduce BC risk in premenopausal women [59], as it may play a role in ovarian folliculogenesis wherein at elevated levels it may reduce follicular estradiol secretion [60, 61]. Thus, postmenopausal female with elevated leptin levels could be at a reduced risk for BC due to leptin resistance with reduced leptin receptor activity [62]. We determined positive correlations of leptin, resistin, and visfatin and negative correlations of adiponectin with most of studied metabolic and anthropometric parameters including BMI, WC, TG, LDLc, and glucose in all studied groups. Also, in postmenopausal controls and cases there were positive correlation of leptin, resistin, and visfatin and negative correlations of adiponectin with TNM, tumor size, LN metastasis, and histological grade. Similarly, it was reported that serum leptin and resistin levels were positively associated with BMI and adiponectin is negatively correlated with BMI [52]. To our knowledge, both visfatin and resistin, as novel markers, have not been studied yet with adiponectin and leptin in relation to other anthropometric and metabolic parameters as risk factors for pre- and postmenopausal BC. Dalamaga et al. [22] hypothesized that visfatin, one of the measured adipokines in our study, by exhibiting proliferative, antiapoptotic, proinflammatory, and proangiogenic properties may be involved in the etiopathogenesis of postmenopausal BC and may represent a missing link between obesity and postmenopausal BC. Visfatin regulates and stimulates the proliferation and DNA synthesis rate of BC cells. It may contribute to BC pathogenesis by augmenting cell proliferation via stimulation of cell cycle progression and by increasing the expression of genes involved in angiogenesis and metastasis. Visfatin activates cell cycle progression by upregulation of cyclins and other cyclin-dependent kinases [63] and increases the synthesis of genes that play a significant role in tumor-related angiogenesis such as vascular endothelium growth factor (VEGF), in metastasis and tumor invasion as matrix metalloproteases [64]. Their finding explains ours regarding visfatin. Visfatin levels were found to be predictor of LN metastasis in postmenopausal BC and were also positively associated with TNM staging and histological grade. On the other hand, resistin similarly may present a molecular link between inflammation and breast carcinogenesis. Expression of resistin was found to be upregulated during monocyte macrophage differentiation, indicating a role for resistin in monocyte–macrophage function [65–67]. In addition, resistin has been shown to exert potent proinflammatory properties by upregulating proinflammatory cytokines, probably via the NF-*κ*B pathway, suggesting a role for resistin in the process of inflammation [68]. In agreement with our results, Kang et al. [20] reported that serum resistin level was found to be significantly higher in the BC group than the control group, after adjustment for possible confounding variables such as BMI and serum glucose. Although resistin was first postulated to contribute to insulin resistance, it has recently been shown that resistin can trigger a proinflammatory state. As the evidence linking inflammation to BC risk grows [69, 70], nevertheless, our results must be interpreted in the context of the study design. When using a cross-sectional design, there is potential for selection bias, particularly in the selection of control groups. Our controls for either pre- or postmenopausal subgroups came from the same study base as our cases with breast cancers regarding age and BMI. Further classification of cases and controls into premenopausal and postmenopausal subgroups was carefully adjusted and exclusion was carried for any suspected subjects to cause any bias. However, important considerations were followed as follows: sample size being sufficient to generate statistically significant associations; the strict criteria for inclusion of case and control participants; the assessment of anthropometric, adipokines, and other parameters made using well-established and validated methods in the same laboratory and in a blinded fashion, which also eliminated uncontrolled confounding from these sources. However, the adjustment for many determinants of serum adipokines levels including inflammatory markers, insulin, and estrogen assessment may be suggested to reinforce the observed associations in our future research.

## 5. Conclusion 

The results suggested a possibility that the serum resistin, leptin, adiponectin, and visfatin levels could be considered risk factors for BC. They could provide a potential link for the association between obesity and BC risk promising to be of diagnostic value for postmenopausal BC.

## Figures and Tables

**Table 1:** The characteristic features, metabolic, and adipokines levels of healthy control and BC group.

	Healthy control	Breast cancer	*P* value
	(*n* = 68)	(*n* = 82)	
Age (years)	52.25 ± 16.62	53.682 ± 13.98	0.567
BMI (kg/m^2^)	24.84 ± 1.35	24.59 ± 1.48	0.062
WC (cm)	76.34 ± 3.05	77.52 ± 3.34	0.026^*^
SBP (mmHg)	124.79 ± 9.21	127.52 ± 10.52	0.096
DBP (mmHg)	83.01 ± 7.98	80.85 ± 9.83	0.147
Glucose (mg/dL)	92.42 ± 12.39	94.80 ± 13.51	0.076
TG (mg/dL)	123.38 ± 27.403	152.60 ± 22.60	0.000^*^
TC (mg/dL)	192.53 ± 17.718	216.52 ± 28.82	0.000^*^
LDLc (mg/dL)	131.43 ± 23.29	145.06 ± 21.32	0.000^*^
HDLc (mg/dL)	45.47 ± 5.41	40.36 ± 4.38	0.000^*^
Leptin (ng/mL)	19.62 ± 2.03	24.59 ± 5.57	0.000^*^
Adiponectin (mg/L)	10.96 ± 1.60	8.44 ± 2.12	0.000^*^
Resistin (*μ*g/L)	22.69 ± 2.58	26.24 ± 1.59	0.000^*^
Visfatin (ng/mL)	15.57 ± 2.41	18.36 ± 3.92	0.000^*^

BMI: body mass index; WC: waist circumference; SBP: systolic blood pressure; DBP: diastolic blood pressure; Tc: total cholesterol; ^*^ *P* < 0.05.

**Table 2:** Anthropometric measurements, metabolic, and adipokines serum levels among all groups.

	Premenopause		Postmenopause	
	Healthy control	Breast cancer	Healthy control	Breast cancer
	(*n* = 27)	(*n* = 44)	(*n* = 41)	(*n* = 38)
Age (years)	35.22 ± 7.59	43.68 ± 6.07^a^	63.46 ± 10.04	65.26 ± 11.34^a^
BMI (kg/m^2^)	23.72 ± 1.26	24.04 ± 1.24	24.22 ± 1.42	24.92 ± 1.51
WC (cm)	76.71 ± 2.91	76.21 ± 3.39	76.09 ± 3.15	79.05 ± 2.59^a^
SBP (mmHg)	123.29 ± 9.31	124.43 ± 7.42	125.78 ± 9.08	131.10 ± 12.42^a^
DBP (mmHg)	77.37 ± 8.0	79.11 ± 9.06	82.73 ± 5.44	86.11 ± 10.40^a^
Glucose (mg/dL)	88.02 ± 10.39	88.80 ± 12.51	98.56 ± 11.68	98.60 ± 12.51
TG (mg/dL)	118.27 ± 29.97	151.50 ± 19.60	126.57 ± 25.37	153.88 ± 26.20
TC (mg/dL)	189.14 ± 23.17	204.52 ± 23.82	194.76 ± 12.718	224.52 ± 30.82
LDLc (mg/dL)	121.56 ± 19.55	142.94 ± 23.27	137.93 ± 23.49	147.52 ± 18.82
HDLc (mg/dL)	44.81 ± 5.37	39.91 ± 4.83	45.90 ± 5.41	40.89 ± 3.78
Leptin (ng/mL)	19.52 ± 2.93	20.32 ± 1.87	19.68 ± 1.19	29.52 ± 4.17
Adiponectin (mg/L)	10.86 ± 1.67	9.92 ± 0.62	11.01 ± 1.58	6.74 ± 1.92
Resistin (*μ*g/L)	24.15 ± 1.24	25.21 ± 1.54	21.73 ± 2.79	27.14 ± 1.79
Visfatin (ng/mL)	15.03 ± 2.91	16.96 ± 1.99	15.26 ± 2.41	21.20 ± 3.62

^a^ *P* < 0.05 as calculated by ANOVA *t*-test versus respective controls (between premenopausal healthy control and premenopausal BC patients and between postmenopausal healthy control and postmenopausal BC patients).

**Table 3:** Pearson's correlation analysis using different variables between all groups and healthy control group.

Variables	BMI	WC	DBP	SBP	Gluc	TG	TC	LDLc	HDLc	Leptin	Adipo.	Resi.
All groups												
BMI	1											
WC	.142	1										
DBP	.112	.073	1									
SBP	-.068-	-.095-	.020	1								
Gluco.	.035	.153	.077	.168^*^	1							
TG	.132	.119	.105	.008	-.018-	1						
TC	.029	-.008-	.026	.020	-.049-	.296^**^	1					
LDLc	.256^**^	.114	.151	-.027-	.162^*^	.214^**^	.306^**^	1				
HDLc	-.074-	.060	.001	-.105-	.074	-.109-	-.210-^**^	.004	1			
Leptin	.313^**^	.276^**^	.187^*^	-.008-	.176^*^	.242^**^	-.052-	.182^*^	-.170-^*^	1		
Adipo.	-.232-^**^	-.295-^**^	-.157-	.023	-.089-	-.377-^**^	-.094-	-.224-^**^	.216^**^	-.595-^**^	1	
Resistin	.051	.051	.052	.192-^*^	.135-	.185^*^	.316^**^	.112	-.281-^**^	.092	-.123-	1
Visfatin	.228^**^	..259^**^	.043	.075	.182^*^	.283^**^	-.032-	.088	-.144-	.549^**^	.506-^**^	.136

Healthy control group												
BMI	1											
WC	-.224-	1										
DBP	.152	-.278-^*^	1									
SBP	-.174-	-.088-	.092	1								
Gluco.	.022	.053	-.017-	-.017-	1							
TG	-.062-	.106	.210	.063	.055	1						
TC	-.129-	-.075-	.426^**^	-.038-	.034	.030	1					
LDLc	.254^*^	-.043-	.076	.121	.079	.176	.120	1				
HDLc	-.007-	.130	-.059-	-.035-	.315^**^	.108	.163	.197	1			
Leptin	-.031-	.120	-.136-	.061	-.072-	-.077-	-.179-	-.003-	.137	1		
Adipone.	-.038-	-.054-	.089	.176	-.115-	.043	-.147-	.065	-.008-	-.002-	1	
Resistin	-.074-	-.076-	-.184-	.012	-.297-	-.220-	-.018-	-.196-	.014	-.215-	.122	1
Visfatin	-.126-	-.201-	-.019-	.015	.050	-.085-	-.014-	-.310-^*^	.085	-.016-	-.024-	-.008-

Adipo.: adiponectin; Gluco.: glucose. ^**^Correlation is significant at the 0.01 level; ^*^correlation is significant at the 0.05 level.

**Table 4:** Correlation of adipokines with TNM stages, tumor size, LN metastasis, and histological grading among postmenopausal and premenopausal BC.

Variables	Leptin	Adipo.	Resistin	Visfatin	TNM	T. size	LN
Postmenopausal BC							
Leptin	1						
Adipo.	-.566-^**^	1					
Resistin	-.435-^**^	-.326-^**^	1				
Visfatin	.627^**^	-.597-^**^	.330^*^	1			
TNM	.338^*^	-.264-^*^	.395^*^	.406^**^	1		
T. size	.241^*^	-.725-^**^	.429^**^	.348^*^	.777^**^	1	
LN	.820^**^	-.742-^**^	.575^**^	.529^**^	.623^**^	.633^**^	1
Grade	.762^**^	-.682-^**^	.509^**^	.132	.475^**^	.780^**^	.413^**^
Premenopausal BC							
Leptin	1						
Adipo.	.283	1					
Resistin	-.143-	-.017-	1				
Visfatin	.060	-.006-	.160	1			
TNM	.204	-.074-	.064	.286	1		
T. size	.062	-.164-	-.081-	.292	.783^**^	1	
LN	.247	-.152-	.010	.177	.626^**^	.580^**^	1
Grade	.083	-.052-	-.039-	.277	.551^**^	.649^**^	.341^*^

^**^Correlation is significant at the 0.01 level; ^*^correlation is significant at the 0.05 level.

**Table 5:** Analysis of multivariate logistic regression with risk for BC as a dependent variable.

	*B*	OR	95% CI	*P* value
Adiponectin	−1.196	0.423	0.132–0.692	0.005
Leptin	0.800	0.244	1.381–3.587	0.001
Resistin	2.121	0.513	3.051–22.790	0.000
Visfatin	0.250	1.089	1.062–1.116	0.001
BMI	0.729	0.370	1.004–4.278	0.049
TG	1.452	6.011	1.823–15.673	0.004

*P* value significant at *P* < 0.05.

**Table 6:** Analysis of multivariate logistic regression with risk for LN metastasis as a dependent variable.

	*B*	OR	95% CI	*P* value
Leptin	0.874	0.742	1.504–2.921	0.003
Resistin	1.452	2.203	4.091–20.791	0.002
Visfatin	1.534	1.080	1.056–1.105	0.003

*P* value significant at *P* < 0.05.
